# A Dual-Threshold Algorithm for Ice-Covered Lake Water Level Retrieval Using Sentinel-3 SAR Altimetry Waveforms

**DOI:** 10.3390/s23249724

**Published:** 2023-12-09

**Authors:** Fucai Tang, Peng Chen, Zhiyuan An, Mingzhu Xiong, Hao Chen, Liangcai Qiu

**Affiliations:** 1 College of Geomatics, Xi’an University of Science and Technology, Xi’an 710054, China; 21210226068@stu.xust.edu.cn (F.T.); 21210061029@stu.xust.edu.cn (M.X.); 21210061019@stu.xust.edu.cn (H.C.); 21210061025@stu.xust.edu.cn (L.Q.); 2 State Key Laboratory of Geodesy and Earth’s Dynamics, Innovation Academy for Precision Measurement Science and Technology, The Chinese Academy of Sciences, Wuhan 430077, China; 3 School of Geodesy and Geomatics, Wuhan University, Wuhan 430079, China; 2023102140024@whu.edu.cn

**Keywords:** satellite altimetry, SAR waveform, lake water levels, lake ice

## Abstract

Satellite altimetry has been proven to measure water levels in lakes and rivers effectively. The Sentinel-3A satellite is equipped with a dual-frequency synthetic aperture radar altimeter (SRAL), which allows for inland water levels to be measured with higher precision and improved spatial resolution. However, in regions at middle and high latitudes, where many lakes are covered by ice during the winter, the non-uniformity of the altimeter footprint can substantially impact the accuracy of water level estimates, resulting in abnormal readings when applying standard SRAL synthetic aperture radar (SAR) waveform retracking algorithms (retrackers). In this study, a modified method is proposed to determine the current surface type of lakes, analyzing changes in backscattering coefficients and brightness temperature. This method aligns with ground station observations and ensures consistent surface type classification. Additionally, a dual-threshold algorithm that addresses the limitations of the original bimodal algorithm by identifying multiple peaks without needing elevation correction is introduced. This innovative approach significantly enhances the precision of equivalent water level measurements for ice-covered lakes. The study retrieves and compares the water level data of nine North American lakes covered by ice from 2016–2019 using the dual-threshold and the SAMOSA-3 algorithm with in situ data. For Lake Athabasca, Cedar Lake, Great Slave Lake, Lake Winnipeg, and Lake Erie, the root mean square error (RMSE) of SAMOSA-3 is 39.58 cm, 46.18 cm, 45.75 cm, 42.64 cm, and 6.89 cm, respectively. However, the dual-threshold algorithm achieves an RMSE of 6.75 cm, 9.47 cm, 5.90 cm, 7.67 cm, and 5.01 cm, respectively, representing a decrease of 75%, 79%, 87%, 82%, and 27%, respectively, compared to SAMOSA-3. The dual-threshold algorithm can accurately estimate water levels in ice-covered lakes during winter. It offers a promising prospect for achieving long-term, continuous, and high-precision water level measurements for middle- and high-latitude lakes.

## 1. Introduction

Rivers and lakes cover approximately 2% of the Earth’s land area, most concentrated in the northern hemisphere [1,2]. Rivers and lakes play essential roles in studying climate change, biodiversity, industrial water use, agricultural irrigation, economic development, and other areas [3,4,5]. In addition, floods threaten social and economic well-being, infrastructure, and human safety, so monitoring water level changes in rivers and lakes is crucial. Traditionally, lake water levels are measured at hydrological stations, but due to the high cost of building and maintenance, the number of stations worldwide has decreased since the 1970s [6,7]. Therefore, a shortage of hydrological stations has resulted in many rivers and lakes worldwide remaining unmeasured [8]. Even in areas with hydrological stations, not all hydrological data is publicly available. Around 286 transboundary rivers and lakes globally account for 60% of the world’s freshwater resources [9]. However, due to political, commercial, and other factors, some hydrological data associated with stations maintained and managed by local governments are deemed sensitive and confidential [10,11]. Satellite altimetry has developed over several decades. Satellite radar altimeters, initially designed to monitor the ocean’s surface, have since been proven helpful for monitoring water levels in inland water systems [12,13,14,15]. Satellite radar altimetry has become a critically important source of data to replace traditional field observations. Altimetry satellites can help overcome the lack of data in many regions of the world and aid in monitoring water levels in inland water systems and transboundary rivers where field data are lacking.

The effectiveness of satellite altimetry in both near real-time and long-term applications has been demonstrated in numerous studies [16,17,18,19,20,21,22,23]. However, many lakes worldwide are in the middle to high latitudes of the northern hemisphere, where ice cover due to cold temperatures during the winter months can significantly affect the hydrological condition of the lakes. The ice cover’s size, location, and duration depend on several factors, including geographic location and climate [24]. However, lake water and ice possess different electromagnetic properties, such as permittivity and electrical conductivity, that have a substantial impact on the interaction between radar signals and the ice surface. Radar waves tend to penetrate ice with lower water content and be reflected, resulting in a stronger reflected signal. Ice with a higher moisture content or in a melting state usually leads to stronger radar signal absorption or attenuation, reducing the radar echo’s amplitude. Additionally, the roughness of the lake ice surface also affects the radar echo. A flat and uniform ice surface usually produces echoes with stronger amplitudes. The distribution and shape of different lake ice types, such as ice ridges, crevasses, and open water areas, introduce complex scattering patterns, causing variations in amplitude and shape [25]. The presence of lake ice results in unreliable estimation of water levels in frozen ice-covered lakes [26,27,28]. Therefore, to obtain continuous and accurate time series of lake water levels, it is critical to address the issue of precise level estimation for ice-covered lakes.

A time series of water levels can be generated by averaging altimeter measurements along the satellite orbit for each cycle on the cross-section of the water body [29]. Estimates of water levels in ice-covered lakes in winter would be improved if open water and lake ice could be distinguished along altimeter trajectories and calculated separately. Moderate resolution imaging spectroradiometer (MODIS) and advanced very high-resolution radiometer (AVHRR) sensor data from optical remote sensing are widely used for lake ice detection in lakes thanks to the daily revisit cycle [30,31,32,33,34], but their lower spatial resolution (up to 1 km) is only applicable to medium to large lakes. In addition, optical remote sensing is impacted by cloud cover and illumination, limiting data availability. Passive microwave remote sensing utilizes generated time series of brightness temperature data to extract lake ice phenology via the thresholding method [35,36,37,38]. In contrast to optical remote sensing, passive microwave remote sensing can work under most meteorological conditions. However, due to its low spatial resolution (3–120 km), it is not suitable for applications on small to medium-sized lakes. It is more suitable for detecting lake ice phenology information on large lakes; in addition, the appropriate choice of threshold value is also very important. The limitations of optical remote sensing and passive microwave can be addressed with active microwave remote sensing. SAR data have been widely used for lake ice phenology extraction in studies over the past few decades [39,40,41,42]. However, the backscatter extracted from SAR images changes complexly during the evolution of lake ice, and its change mechanism is still controversial. The current altimetric satellites used for lake ice monitoring are the Jason series, CryoSat-2, and Sentinl-3A [28,43,44,45]. Compared to other methods, radar altimeters can not only estimate water levels but also be used to detect lake ice.

Beckers et al. [44] used CryoSat-2 waveform data combined with waveform characteristics, peak intensity, and adjacent waveform ratio to distinguish lake ice from open water, then estimated the thickness of lake ice and compared it with in situ data. The root mean square error (RMSE) is less than 0.33 m. However, the accuracy of this distinction is also affected by other factors, such as the physical properties of the lake ice (such as air bubble content and roughness), atmospheric conditions, and satellite data processing. Duguay et al. [46] found that the backscatter coefficient of radar altimeters at rivers or lakes varies seasonally. Kang et al. [37] confirmed that the temporal variation of brightness temperature is a sensitive indicator for detecting ice sheet formation. Howell et al. [47] used the QuikScat Ku-band threshold method for the backscatter coefficient to extract phenology information about lake ice. Shu et al. [28] used brightness temperature to extract lake ice phenological information, and the results were consistent with the freezing and breakup dates from field observations. However, relying on a single brightness temperature or backscatter coefficient for assessing lake ice phenology information may result in an accuracy that is too dependent on the chosen threshold values. Open water and lake ice can be distinguished more reliably if two thresholds are combined. Li et al. [48] proposed a model based on the Ku-band scattering coefficient to determine whether the lake is in the ice-covered period and proposed a method to invert the lake ice thickness, which has been verified using field data and has an accuracy of 0.2 m. However, the pulse-Doppler-limited waveform of the SAR altimeter is different from the pulse-limited waveform, and whether it is applicable remains to be studied.

When a lake becomes covered by ice, the waveform acquired using satellite altimeters changes, leading to unreliable estimates of water levels using existing retracking methods [26,28,49]. Therefore, developing a new retracking algorithm is crucial to obtain continuous and accurate water level time series for ice-covered lakes, which is essential for various applications such as climate research and water resource management. Shu et al. [28] proposed a bimodal correction algorithm for calculating observed data water levels in Sentinel-3 affected by lake ice, which was shown to provide reliable water levels for lakes covered by lake ice in winter. Shu et al. [28]’s proposed method, which uses the maximum point rather than the mid-height point of the first leading edge as the retracking point to calculate the ice-top surface elevation, requires an additional calculation of the systematic deviation of the maximum point from the mid-height point of the four SRAL SAR retracking algorithms. Ziyad et al. [50] used a k-means clustering method to define a threshold to differentiate between water and ice, separating open water and ice-covered areas in the Jason-2 observation data. Water level series were derived using only observation data from open water without interference from lake ice. However, the obtained water level time series were not continuous and lacked water level information when the lake surface was frozen. Yang et al. [51] tested several threshold retracking algorithms and proposed an improved sub-waveform threshold retracking algorithm for T/P and Jason’s dual peak waveforms. This algorithm improved the accuracy of water level estimation in ice-covered lakes, particularly during winter when abnormal water levels are prevalent. However, further research is necessary to determine its suitability for Sentinel-3 waveforms.

This article presents a dual-threshold tracking algorithm, which provides a reliable method for water level observation of lakes covered by ice in mid to high-latitude regions. To accurately identify lake ice and determine the freezing and thawing dates, the proposed method uses active radar altimeters and passive microwave radiometers to provide backscatter coefficients and brightness temperatures. When the lake surface type is identified as ice, the dual-threshold algorithm is employed to calculate the ice cover surface and bottom threshold points based on the bimodal waveform when the surface is covered by ice. Then, the precise tracking point is calculated using the 50% threshold interpolation to determine the thickness of the lake ice. Finally, the equivalent water level height of the frozen lake is inverted. The method in this paper compared to Shu et al. [28] and Beckers et al. [44] uses the mid-height of the leading edge as the retracking point without additional calculations and clarifies the method of identifying the judgment of the lake ice without the aid of temperature.

The article is organized as follows: Section 2 presents the study area and database. Section 3 describes the principle of satellite altimetry and proposes a method for determining the current lake surface type. A dual-threshold algorithm for retrieving the water level of lakes covered with ice is also introduced. Section 4 compares the water levels obtained using several retrackers with in situ measurements of nine lakes with different lake surface types. The influence of lake ice on SAR waveform retracking is studied, and the performance of the dual-threshold algorithm is evaluated. Section 5 summarizes the method and presents the conclusion.

## 2. Study Area and Databases

### 2.1. Study Area

**Figure 1:**
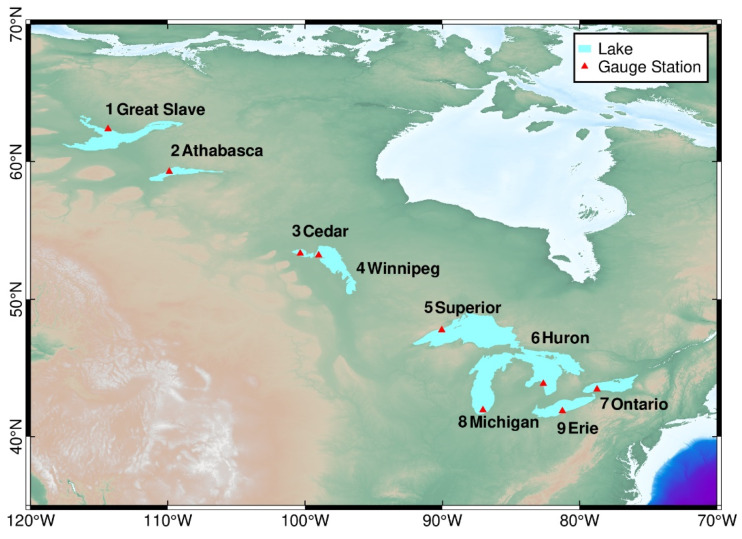
Geographical distribution of lakes and hydrological stations in the study area.

The study area of this article consists of nine freshwater lakes in Canada and the United States, whose geographical distribution is shown in Figure 1. The lakes cover the entire territory of North America, not only with large latitudinal spans but also with significant differences in lake areas. Great Slave Lake, Lake Athabasca, Cedar Lake, and Lake Winnipeg in Canada are at middle and high latitudes (52° N–63° N), where the climate of the lakes is severe in winter, and the lakes are completely covered with ice. Among them, Great Slave Lake is the second largest lake in Canada, covering an area of more than 27,000 km^2^. It is at the highest latitude in the study area and is covered with ice for an average of 6 months per year. The Great Lakes are all located at middle latitudes (41° N–48° N), and their winter icing conditions vary, mainly depending on winter temperatures [52]. Lake Superior is the second largest lake by area and the largest freshwater lake by volume in the world, with an area of over 80,000 km^2^ and a water storage capacity of 12,100 km^3^. The winter ice cover conditions of the study lake were determined by combining a visual interpretation of Landsat-8 remote sensing imagery with ice data from the Canadian Cryosphere Watch (https://ccin.ca/ccw/lakeice/current/monitoring, accessed on 25 June 2022). Table 1 lists the geographic feature data of the study lakes, winter icing conditions, and Sentinel-3 ground track information covering them. The ground trajectory was determined using the satellite’s orbit, the geographical location, and the area of the lake. This article selects only two trajectories near the hydrological station for the nine lakes.

### 2.2. Database

#### 2.2.1. Altimetry Data

In March 2016, the European Space Agency (ESA) launched the Sentinel-3A satellite equipped with advanced instruments to systematically measure Earth’s oceans, land, ice, and atmosphere to detect and understand global-scale Earth system processes. The Sentinel-3 carries an SRAL, a fully redundant dual-frequency, nadir-looking radar altimeter. The Ku band (13.575 GHz) is used for measuring surface heights, while the C band (5.41 GHz) is used for ionospheric correction [53]. SRAL has two primary operating modes: low-resolution mode (LRM) and synthetic aperture radar (SAR) mode. LRM is used only for testing during the commissioning phase. Due to the coherence between pulse reflections and Doppler shifts, SAR mode significantly improves along-track resolution (~300 m), effectively reducing terrestrial contamination in radar signals and making it possible to estimate water levels in small and medium-sized lakes [54]. In the context of SAR altimetry, the small footprint allows us to disregard the minimum point effect [55,56,57]. More importantly, it eliminates signal contributions before and after the nadir point, making it feasible for use in near-coastal regions with less influence from land pollution on height measurements compared to LRM [55,57]. Sentinel-3 has a 27-day operational cycle and a highly inclined polar orbit (98.5°), providing global altimetry coverage up to 81.35° of latitude. It is the world’s first altimetry SAR model with global coverage. It has good global coverage and sufficient spatial and temporal resolution and is an ideal tool for monitoring the water levels of inland lakes. Sentinel-3 also carries a passive microwave radiometer (MWR), a two-channel passive microwave system operating at 23.8 GHz and 36.5 GHz to measure atmospheric water vapor and liquid water content for correcting atmospheric attenuation caused by the moist troposphere [53]. Furthermore, MWR simultaneously measures the lake surface brightness temperature on the two channels for detecting lake ice.

ESA provides two levels of Sentinel-3 data. Level 1 contains orbit information and echoes the altimeter receives, also known as waveforms. Level 2 contains various geophysical corrections and height estimates. This article uses the enhanced data file from 20 Hz Level 2 for the non-time-critical (NTC) period from June 2016 to October 2019 in SAR mode, provided by the ESA Copernicus Open Access Hub (https://scihub.copernicus.eu/, accessed on 1 May 2022).

#### 2.2.2. In Situ Water Level

Measured water level data from lake hydrological stations were obtained from Environment and Climate Canada (https://wateroffice.ec.gc.ca/, accessed on 1 June 2022) and the NOAA Centre for Operational Oceanographic Products and Services Centre (https://tidesandcurrents.noaa.gov, accessed on 1 June 2022). Detailed information on the hydrological station can be found in Table 2. The water level gauges used at the hydrological station are a float-actuated system and pressure-actuated systems. When lakes are frozen, the water level meters can measure the equivalent water level using heating equipment that melts the ice or via pressure sensors [58]. The vertical datum referenced by the Great Lakes hydrological station measurements is the International Great Lakes Datum, 1955 (IGLD 1955), which can be converted to the EGM2008 geodetic reference level using the vertical datum conversion tool VDatum, provided by NOAA (https://vdatum.noaa.gov/docs/datums.html, accessed on 20 June 2022). The vertical datum referenced by hydrological stations in Canada is the Canadian Geodetic Vertical Datum of 1928 (CGVD28), which cannot be converted to the Earth Gravitational Model 2008 (EGM2008) due to a lack of crucial conversion information. Therefore, to make comparisons, it is necessary to determine the systematic deviation, i.e., the average deviation between the data measured at the hydrological station and the water estimated by the altimeter.

#### 2.2.3. Water Mask

The Global Lakes and Wetlands Database (GLWD) was created by the World Wildlife Fund (WWF) and the Center for Environmental Systems Research at the University of Kassel, Germany. Three levels of the database have been generated by combining the best available resources on lakes and wetlands globally with GIS capabilities. Among them, level 1 (GLWD-1) not only includes data on the 3067 largest lakes (area ≥ 50 km^2^) and 654 largest reservoirs (storage capacity ≥ 0.5 km^3^) in the world but also features a rich collection of attribute data on these water bodies. In this article, the GLWD-1 lake boundary polygons in the database are used as masks to extract SARL altimetry values of the lakes in the target area. The shapefiles are available from the WWF website (https://www.worldwildlife.org/, accessed on 1 June 2022).

## 3. Principles and Methods

### 3.1. Principles

As a moving platform, the satellite uses the microwave radar altimeter to transmit microwave pulses with specific pulse frequencies to the Earth via the antenna. As a result, the water surface reflects part of the incident radar wave, the satellite receiver accepts the echo signal reflected from the water surface, and the round-trip time of the radar pulse wave is accurately recorded. Then, the theoretical vertical distance R between the satellite and the water surface can be calculated by:

(1) $$R=\frac{1}{2}c\times t$$

where c is the speed of light (299,792,458 m/s), and t is the time interval for the round trip. The atmospheric influence affects the water level estimation of the altimetry due to the delay of the radar pulse wave. In addition, since the altimetry water level is the average distance from the satellite to within the footprint, tides, and atmospheric pressure can also influence it [59]. Since the lake is enclosed and relatively small compared to the ocean, the effects of ocean tides, tidal loads, reverse air pressure, and sea state deviations are not considered. Therefore, the water level of the lake is calculated as follows [14]:

(2) $$h=H-R_{\mathrm{cor}}-\left(R_{\mathrm{iono}}{+R}_{\mathrm{dry}}{+R}_{\mathrm{wet}}{+R}_{\mathrm{pole}}{+R}_{\mathrm{solidearth}}\right)-N_{\mathrm{geoid}}$$

(3) $$R_{\mathrm{cor}}{=R+R}_{\mathrm{retrk}}$$

(4) $$R_{\mathrm{retrk}}=\Delta G\times c\times \left(C_{\mathrm{retrk}}-C_{\mathrm{ntp}}\right)$$

where H is the height of the satellite relative to the reference ellipsoid World Geodetic System 1984(WGS84); $R_{\mathrm{cor}}$ is the tracker range given by the nominal tracking point on the waveform, from the satellite to the water surface by a specific retracker; $R_{\mathrm{reetrk}}$ represents the relative distance between the retracking point and the nominal retracking point; and ΔG is the time interval between two adjacent bins in the waveform (for Sentinel-3, ΔG=3.125 ns). $C_{\mathrm{retrk}}$ and $C_{\mathrm{ntp}}$ are the distances in units of bin numbers from the first bin of the waveform window to the nominal tracking position and the retracking position, respectively (for Sentinel-3, $C_{\mathrm{ntp}}=43$). $R_{\mathrm{iono}}$, $R_{\mathrm{dry}}$, $R_{\mathrm{wet}}$, $R_{\mathrm{pole}}$, and $R_{\mathrm{solidearth}}$ are the corrections for range biases caused by the ionosphere, dry troposphere, wet troposphere, polar tides, and solid Earth tides, respectively, and $N_{\mathrm{geoid}}$ are the corrections between the geoid EGM2008 relative to the reference ellipsoid WGS84 [35]. In this article, the EGM2008 geoid calibration provided in NOAA’s VDatum tool (v3.4) is used to match the geoid model referenced by the hydrographic station measurements.

The Sentinel-3 SRAL Level 2 “enhanced” data provides H, $R_{\mathrm{cor}}$, and geophysical corrections (Table 3). Two types of ionospheric corrections are included in the Level 2 product: the dual-frequency estimation correction (Ku and C bands) and the model correction. Since the dual-frequency ionospheric correction may be hindered by land contamination of the C-band signal, this article uses the ionospheric correction derived from the Global Ionospheric Map (GIM) model [60]. This article uses the global ionospheric model (GIM) to calibrate the ionospheric delay. Two tropospheric correction products are also available for Level 2 data: MWR microwave radiometer measurements for calculating the wet troposphere and the European Centre for Medium-Range Weather Forecasts (ECMWF). This model correction was chosen because the upland contaminates the radiometer [61]. The tropospheric correction provided by the ECMWF is further divided into sea-level correction and lake surface correction [60]. Since the tropospheric correction is related to the surface height, the lake surface correction is chosen.

### 3.2. Lake Surface Type Identification

In this article, we use a combination of altimeter backscatter coefficient (Sig0) and passive microwave radiometer (MWR) brightness temperature data (TB-23.8, TB-36.5) collected at two frequencies to detect and identify lake ice. The simultaneous collection of active and passive microwave data from the same satellite can significantly enhance data analysis capabilities, ensure the perfect coordination of the two payloads [62], and improve lake ice identification accuracy.

When the lake surface freezes in winter, the backscattering coefficient and brightness temperature change periodically, dividing the lake-freezing process into four stages. In the first stage, the lake temperature gradually drops below zero at the beginning of winter. At this stage, river icing begins by forming a fine floating ice layer along the riverbanks. In lakes during freezing, floating ice prevents the development of wind-induced waves, thereby increasing the specular reflection of the water surface [26]. This causes a gradual increase in the backscattering coefficient until a complete and continuous ice cover is generated. The last peak of winter in the backscatter coefficient is the freezing point of lake ice [45]. In the second stage, with the gradual growth of the lake ice, the volume scattering of the altimeter scattering signal within the lake ice increases, leading to a rapid decrease in the backscatter coefficient [63]. Currently, the gradually thickening lake ice reduces the influence of the liquid water under the ice. It releases its microwave energy, increasing the brightness temperature above the water body [64]. In the third stage, as it warms up in early spring, the lake ice deteriorates under the influence of solar radiation (tree-like air channels form directly beneath the ice), at which point a decrease in backscatter can be observed. This is due to the following reasons: the ice begins to melt, creating pools of liquid water, the presence of which increases the dielectric constant and thus signal absorption, in addition to the fact that the accumulation of water on the surface causes the radar signal to undergo specular reflection, which also leads to a decrease in the intensity of backscattering [46,65]. Finally, in stage IV, as the temperature rises, the shortwave radiation absorption on the ice surface increases, and the brightness temperature increases. After the lake ice melts, a layer of the ice-water mixture forms on the surface, which causes mirror reflection and hinders the penetration of radar waves. Surface scattering dominates, increasing the backscatter coefficient until the lake ice is wholly ablated [66]. After that, the backscatter coefficient and brightness temperature undergo a complete ice cycle change.

**Figure 2:**
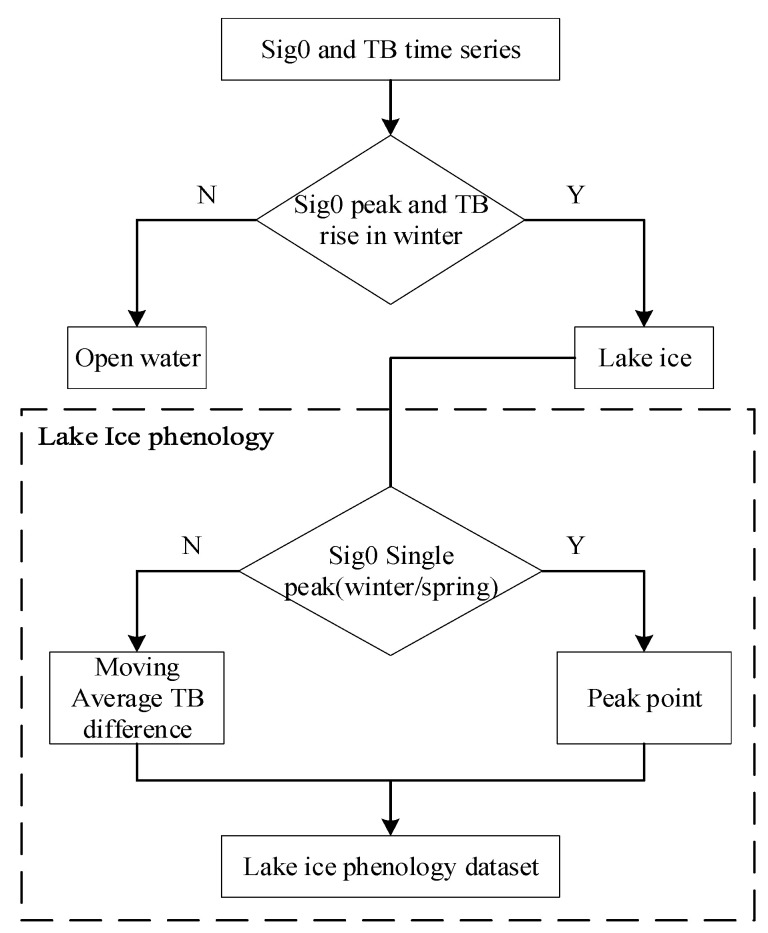
Judgment of Lake Ice using Backscattering Coefficient and Brightness Temperature Detection.

This article tests a modified method that combines the backscatter coefficient and brightness temperature to determine the current lake surface type (see Figure 2). The last peak of each year in the backscatter time series marks the lake ice, while the initial peak in spring means that the lake ice has melted. The data within these two time points indicate that the lake’s surface is currently covered by lake ice. If there are multiple or no peaks in a short period, it is necessary to introduce the brightness temperature difference based on the 23.8 and 36.5 GHz channels and use the moving average brightness temperature difference to determine the freezing and melting dates. Taking advantage of the significant difference in brightness temperature between lake water and lake ice in the study area and the long period of lake ice, the average difference in brightness temperature between the current day and the two before and after that meets the threshold condition is searched in the brightness temperature time series. The freezing and melting day of the ice was determined based on the occurrence and disappearance of high brightness temperatures [67].

(5) $$\begin{array}{l} \mathrm{Min}\left(\mathrm{Mean}\left({\mathrm{Tb}}_{i}{,\mathrm{Tb}}_{i-1}{,\mathrm{Tb}}_{i-2}\right)-\mathrm{Mean}\left({\mathrm{Tb}}_{i+2}{,\mathrm{Tb}}_{i+1}{,\mathrm{Tb}}_{i}\right)\right)\\ {\mathrm{and}\;\mathrm{Tb}}_{i}>200\;K\; \end{array}$$

(6) $$\begin{array}{l} \mathrm{Max}\left(\mathrm{Mean}\left({\mathrm{Tb}}_{i}{,\mathrm{Tb}}_{i-1}{,\mathrm{Tb}}_{i-2}\right)-\mathrm{Mean}\left({\mathrm{Tb}}_{i+2}{,\mathrm{Tb}}_{i+1}{,\mathrm{Tb}}_{i}\right)\right)\\ {\mathrm{and}\;\mathrm{Tb}}_{i}>200\;K\; \end{array}$$

where *Max*(), *Min*(), and *Mean*() are the maximum value, minimum value, and average value, respectively. *Tb* is the brightness temperature time series, *Tb_i_* is the average value of the two frequency brightness temperatures at the time i (where the brightness temperature *Tb_i_* > 200 K), and Tb_i±1_ and *Tb_i±_*_2_ are, respectively, the brightness temperature averages of two frequencies at 1 and 2 moments adjacent to the moment i in the brightness temperature time series. This method takes advantage of the large difference in brightness temperature between lake water and lake ice. Equation (5) represents the day when the sliding brightness temperature difference is the smallest, that is, the day when the brightness temperature changes from low to high and reaches a high brightness temperature value. The corresponding date is the day when the lake ice freezes. Equation (6) indicates that it is the day when the sliding brightness temperature difference is the largest, that is, the day when the brightness temperature goes from high to low and leaves the high level, and the corresponding date is the day when the lake ice melts.

### 3.3. Dual-Threshold Retracking Algorithm

**Figure 3:**
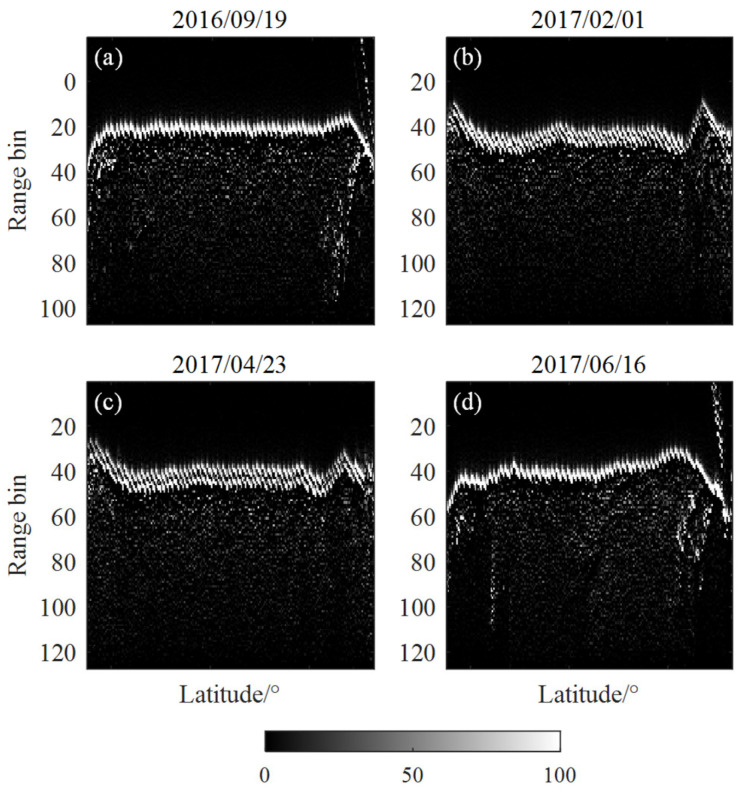
Setinel-3 SAR waveform data from different dates on Great Slave Lake. The fringe associated with the single backscattering of the radar echoes due to the open water is visible (**a**,**d**). The fringe associated with the double backscattering of the radar echoes due to the ice is visible (**b**,**c**).

The altimetry radar waveforms show specific features on snow-covered lakes. For example, for the Sentinel-3 Ku-band waveforms, a stepped break appears at the waveform leading edge due to double backscattering from the presence of ice. Figure 3 shows the waveform overlays at Great Slave Lake on 19 September 2016, 1 February 2017, 23 April 2017, and 26 June 2017, respectively, representing the lake surface covered with open water, lake ice, lake ice, and open water, respectively. Where the columns in each subplot represent waveforms collected during a cycle along the latitudinal direction, the color gradient indicates the change in the power difference between adjacent bins of the waveform, with bright spots representing an increase and dark spots representing a decrease or maintenance of waveform power. Only one distinct bright band in Figure 3a,d corresponds to the lake surface signal from open water. In Figure 3b,c, the presence of two distinct bright bands representing the two leading-edge stages corresponds to the reflected signals from the surface and bottom of the lake ice, with the spacing of the two bright bands in Figure 3c being larger than the spacing of the bright bands in Figure 3b due to the thickening of the lake ice over time.

**Figure 4:**
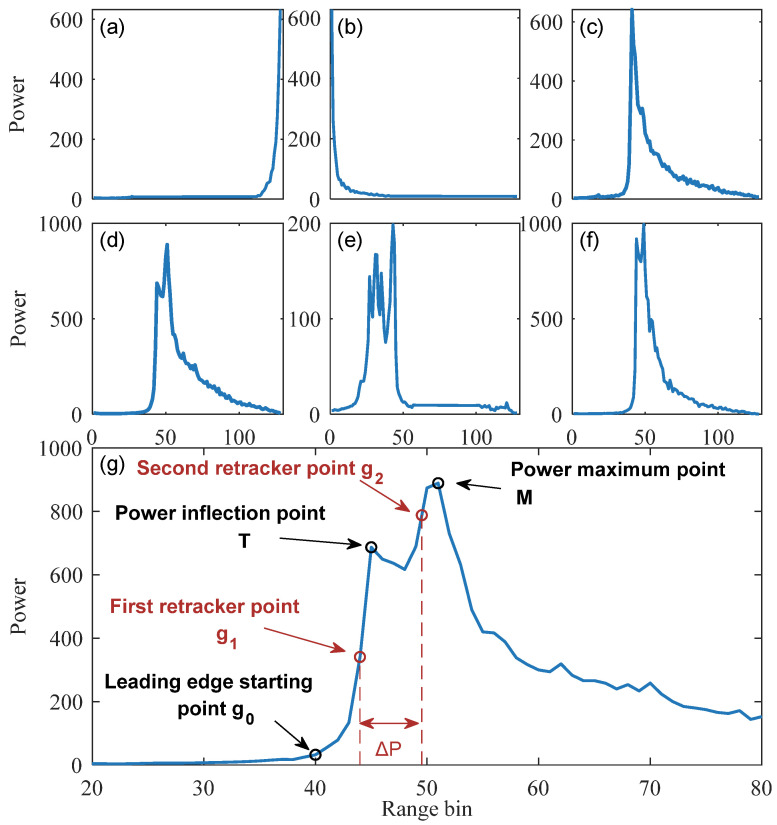
Several waveforms from Sentinel-3 SAR mode: (**a**) waveforms with peaks appearing too late, (**b**) waveforms with peaks appearing too early, (**c**) waveforms generated by open water, (**d**) double-peaked waveforms generated by lake ice, (**e**,**f**) multi-peaked waveforms caused by lake ice, (**g**) Typical bimodal waveform (To highlight the leading edge power variation, only bin values between 20 and 80 power are shown).

Figure 4 shows several different waveforms under different lake conditions. When the lake surface is covered with ice, the shape of the SRAL SAR waveform changes significantly, and the returned waveform changes from a single-peaked waveform to a double-peaked waveform or even a multi-peaked waveform. The multi-peaked waveform may be caused by impurities or air bubbles in the ice. The double-peaked or multi-peaked waveform disables the standard SRAL SAR retrackers and identifies the wrong retracing point, resulting in the estimated lake level being low compared with the results of the hydrological stations. Beckers et al. [44] found two peaks at the leading edge of the waveform and attributed the first peak to the reflection of the radar signal at the ice surface (i.e., the air/ice interface). The second peak was the reflection from the ice bottom (i.e., ice/water interface). The difference between the two peaks (ΔP) is related to the ice thickness, and the peak power point is identified as the retracking point in the waveform. Since the distance difference between different bins is an integer multiple of the distance resolution, this would make the individual waveform estimations of the lake ice thickness a discrete estimate. The waveforms along the track were averaged to obtain a continuous estimate of lake ice thickness, but this significantly reduced the spatial resolution of the estimate. Shu et al. [28] proposed a bimodal correction algorithm to assess whether the lake surface is covered with ice. However, it requires the calculation of additional systematic deviations, and the algorithm is ineffective in determining when there are multiple peak cases. 

**Figure 5:**
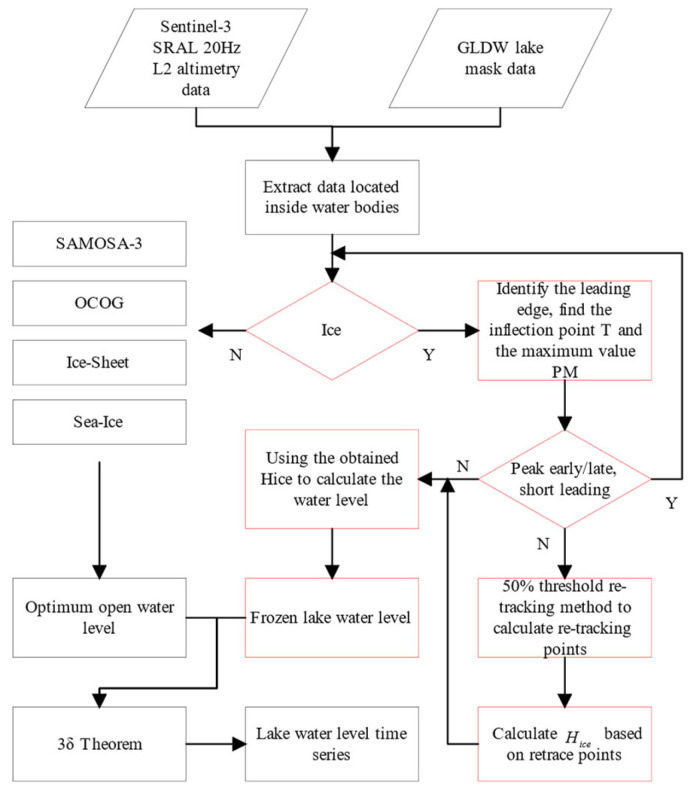
The flowchart of the double threshold algorithm and the red part is the improvement compared to the original algorithm (The 3σ guideline refers to the elimination of roughness using three times the median error).

In this article, proposing a double-threshold algorithm based on the bimodal correction algorithm, which does not require the additional calibration of system bias and can correctly identify waveforms with multiple peaks, overcoming the shortcomings of the double-peak correction algorithm. The algorithm’s core is finding the retracking point of the two peaks in the waveform. First, the starting point $g_{0}$ of the leading edge in the waveform is determined, and then the inflection point T of the power within the leading edge window [$g_{0},g_{0}+20$] is determined. If the inflection point is located near the middle of the leading edge, it indicates that the waveform may consist of signals from two backscattering surfaces (water and ice). The waveform is rejected if the inflection point is near the top of the leading edge. The thresholds for the ice surface and ice bottom signals in the waveforms are calculated based on the inflection points. Then the retracking points of the waveforms $g_{1}$ and $g_{2}$ are interpolated to calculate the ice thickness [44]. The specific calculation method is shown in Equation (9). Finally, the equivalent water level of the ice-covered lake is obtained from Equation (7). Figure 5 shows the flowchart of continuous water level estimation.

(7) $$h=H-R_{\mathrm{cor}}^{\prime }-\left(R_{\mathrm{iono}}{+R}_{\mathrm{dry}}{+R}_{\mathrm{wet}}{+R}_{\mathrm{pole}}{+R}_{\mathrm{solidearth}}\right)-N_{\mathrm{geoid}}-\left(\rho_{\mathrm{water}}-\rho_{\mathrm{ice}\;}\right){\times H}_{\mathrm{ice}\;}$$

(8) $$R_{\mathrm{cor}\;}^{\prime }{=R+R}_{\mathrm{ice}}$$

(9) $$H_{\mathrm{ice}}=\frac{1}{2}\times \Delta G\times \frac{c}{n_{\mathrm{ice}}}\times \Delta P$$

where $\rho_{\mathrm{water}}$ (1000 kg/m^3^) and $\rho_{\mathrm{ice}}$ (917 kg/m^3^) denote the densities of water and ice, respectively, $R_{\mathrm{ice}}$ represents the relative distance between the retracking point of the first wave crest of the waveform formed by the air/ice surface and the preset tracking point. $R_{\mathrm{cor}\;}^{\prime }$ is the retracking distance from the satellite to the ice surface, and $H_{\mathrm{ice}}$ represents the thickness of the ice layer. $n_{\mathrm{ice}}$ signifies the refractive index of ice, and the refractive index of the Ku-band pulse wave in the ice is 1.7861 [68]. ΔP is the bin value spacing of the retracking points between the two wave peaks, and the rest of the parameters are the same as those defined above.

The main steps for estimating the water level of a lake covered with lake ice from the SRAL altimeter are as follows:

In the first step, the brightness temperature and backscatter coefficient are used to determine the type of lake surface, as described in Section 3.2. When the lake surface is covered with ice, the following steps identify the starting point ($g_{0}$) of the leading edge of the waveform and the inflection point (*T*) within the leading-edge window. First, one must calculate the adjacent power difference, *d_i_*, of the waveform and its standard deviation, *S*. When the first occurrence of the adjoining power difference, di, being greater than 0.2×S happens, the corresponding bin value as the starting point of the leading edge $g_{0}$ is recorded. Then, the leading-edge window is reduced to [$g_{0}{,\;g}_{0}+20$], and the maximum power $P_{M}$ is found in the leading-edge window at point *M*. When di in the leading-edge window is continuously smaller than $d_{i+1}$, it is recorded as the inflection point T.

(10) $$d_{i}{=P}_{i+1}-P_{i}$$

(11) $$S=\mathrm{std}\left(d_{1}{,d}_{2},\ldots {,d}_{i-1}\right)$$

In the second step, to filter the waveform and reduce the impact of noise, the following types of waveforms need to be removed: waveforms with shorter leading edges (satisfying $M-g_{0}<3$) are eliminated, because these waveforms only record background noise or a small part of the leading edge. Waveforms whose peaks appear too early or too late (*satisfying*10>M or 118<M) are eliminated because the waveform cannot capture the complete peak (as in Figure 4a,b). Waveforms with multiple peaks in the leading edge where the power difference between the peak and the adjacent previous trough is less than $0.1\times P_{M}$ (Figure 4e), and waveforms with inflection points near the peak ($P_{T}>0.9\times P_{M}$) are also discarded (Figure 4f).

In the third step, the first sub-waveform is defined as [$P_{g_{0}}{,\;P}_{T+1}$] and the second sub-waveform as [$P_{T}{,\;P}_{M}$]. Then the power threshold (${\mathrm{Th}}_{1}$) of the retracking point corresponding to the ice surface is calculated using the approximate 50% threshold retracking algorithm [69], with $P_{g0}$ representing the power at the start point of the leading edge. Afterward, $P_{T}$ is taken as the average power of the trailing edge of the first waveform and the power threshold ${\mathrm{Th}}_{2}$ of the retracking point corresponding to the ice bottom is calculated.

(12) $${\mathrm{Th}}_{1}=0.5\times \left(P_{T+1}{+P}_{g_{0}}\right)$$

(13) $${\mathrm{Th}}_{2}=0.5\times \left(P_{T}{+P}_{M}\right)$$

In the fourth step, the bin corresponding to the first power greater than the threshold Th in the main peak is identified as k in the two sub-waveform. The retracking points ($g_{r}$) are obtained using interpolation between the bin k and the bin before it (k−1) as shown in Equation (14). The difference, ΔP, between the two retracking points $g_{2}$ and $g_{1}$ obtained via interpolation is substituted into Equation (7) to obtain the water levels of frozen lakes in winter [69].

(14) $$g_{r}=k-1+\frac{{\mathrm{Th}}_{r}-P_{k-1}}{P_{k}-P_{k-1}}$$

## 4. Result and Discussion

The performance of each SRAL SAR retracker in the retrieval of lake water level is evaluated with three indicators: the Pearson’s correlation coefficient (R) between the estimated water level and the measured value of the retracking algorithm, the deviation (bias), and the root mean square error (RMSE).

### 4.1. Performance of Retracking Algorithms under Different Ice Cover Conditions

**Figure 6:**
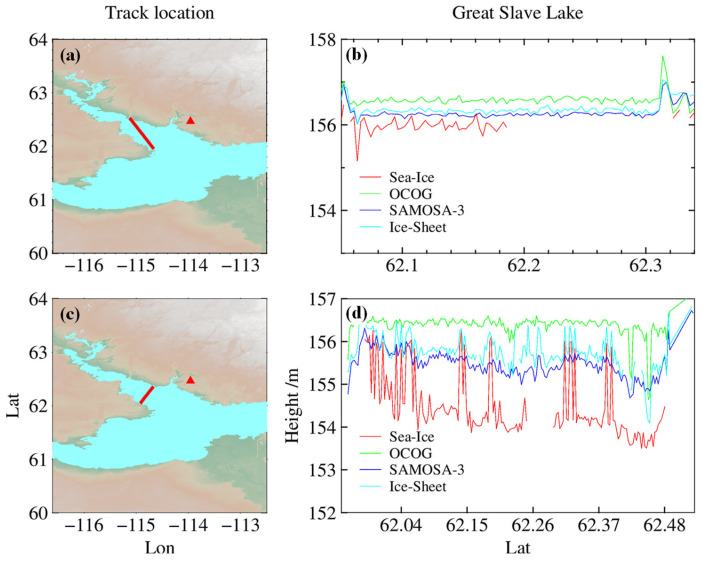
Comparison of ground tracks and water levels across Great Slave Lake on 27 September 2017 and 13 March 2018. (**a**,**c**) Sentinel-3 ground track; (**b**,**d**) Water level comparison. The red triangle is the water level station, and the strip is the ground track.

The algorithm proposed in this article only applies when the lake is covered with ice. However, the standard retracking algorithm is still used for water level estimation at other times, so evaluating the accuracy of several standard retracking algorithms is necessary. Sentinel-3 has four standard retracking algorithms for estimating water level in SAR mode: OCOG for sea ice margins [70], SAMOSA-3 for ocean surface [71], ice sheet for ice cover [72], and sea ice [73]. Figure 6 shows the ground tracks of tracks 37 and 681 that crossed Great Slave Lake on 27 September 2017 and 13 March 2018 and compares the water levels obtained using different retrackers. As seen from Figure 6b,d, the sea ice re-tracker has a high data loss rate when retrieving water levels in open waters and large data fluctuations when retrieving water levels in frozen lakes, so subsequent analysis will not be discussed later.

**Figure 7:**
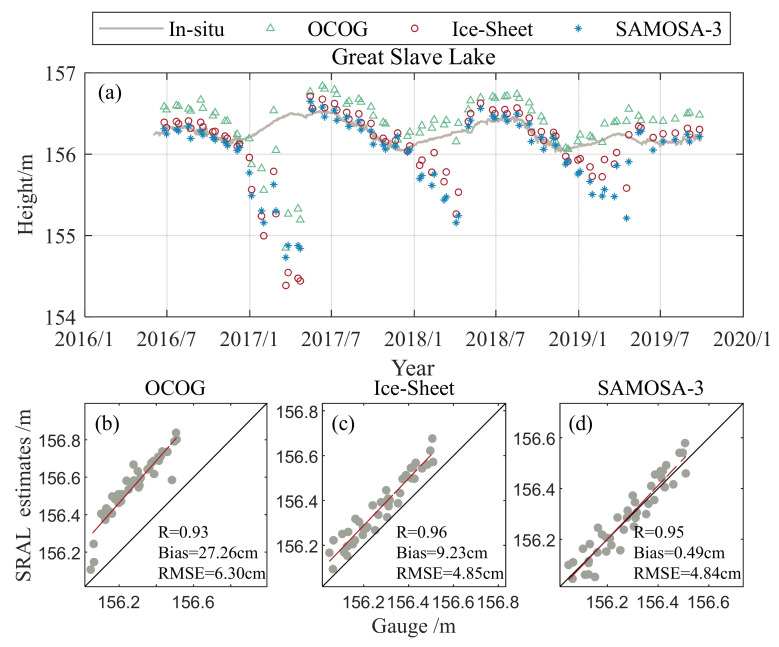
Comparison of the water level measured using the retracker in Great Slave Lake from 2016 to 2019 with the hydrological station and the correlation between the water level obtained using the retracker and the in situ gauge water level. (**a**) Comparison of estimated water levels for each retrackers, (**b**) OCOG, (**c**) ice sheet, (**d**) SAMOSA-3.

The accuracy of the remaining three SRAL SAR retrackers is evaluated to study the accuracy of the water level estimation of different algorithms under different lake surface conditions. Since Great Slave Lake is entirely covered with ice in winter, it is discussed in this section. Figure 7a shows the time series of water levels estimated using the three standard retracking algorithms and the in situ measurements from 2016 to 2019. The results indicate that the three standard retracking algorithms are less accurate in estimating water levels when the lake surface is covered with ice in winter. However, compared with the measured water level, the water level estimation is more consistent in other seasons, and the three algorithms only apply to the ice-free period. Therefore, only the accuracy of three standard retracking algorithms in the ice-free condition of the lake is analyzed in this section. Figure 7b–d shows the correlation plots between the water levels estimated using the three retracking algorithms and the measured water levels during the ice-free period. The bias of OCOG, ice sheet, and SAMOSA-3 are 27.26 cm, 9.23 cm, and 0.49 cm, respectively; the correlation coefficients (R) are 0.93, 0.96, and 0.95, respectively; the RMSE are 6.30 cm, 4.85 cm, and 4.84 cm, respectively. The results show that the water levels in open water obtained using the three retrackers have a high degree of agreement compared with the measured values. Among the three algorithms, SAMOSA-3 exhibits the highest accuracy in estimating water levels in open water.

Table 4 shows the analysis of nine lakes compared to the measured values for different tracks with different lake types. Great Slave, Athabasca, Cedar, Winnipeg, and Erie are partially or entirely covered with ice in winter. Therefore, each lake has two sets of statistics for periods with and without ice cover. The accuracy of the three heavy trackers for Great Slave Lake, Lake Athabasca, Cedar Lake, Lake Winnipeg, and Lake Erie improved after excluding water levels acquired during periods of lake ice. Especially in Great Slave Lake, the R of all three retrackers improved by about 80%, and RMSE was reduced by 83%, 91%, and 89%, respectively. Due to an accurate hydrological station elevation datum, this section mainly considers the bias of the retrackers in the Great Lakes. The mean bias of the OCOG, Ice sheet, and SAMOSA-3 in the Great Lakes is 33.70 cm, 9.94 cm, and 2.61 cm, respectively. The mean RMSE of OCOG, Ice sheet, and SAMOSA-3 retracker were 4.85 cm, 3.98 cm, and 4.28 cm, respectively. The results are consistent with the description in Shu et al. [28]. When the lake surface is not frozen, the three standard retracking algorithms can reasonably estimate the lake water level. When the lake surface is frozen, the three standard retracking algorithms cannot obtain reliable water levels, so it is necessary to study an algorithm suitable for the ice period. The three retracking algorithms have similar accuracy levels, with SAMOSA-3 closest to the measurements.

### 4.2. Lake Surface Type Identification Verification

**Figure 8:**
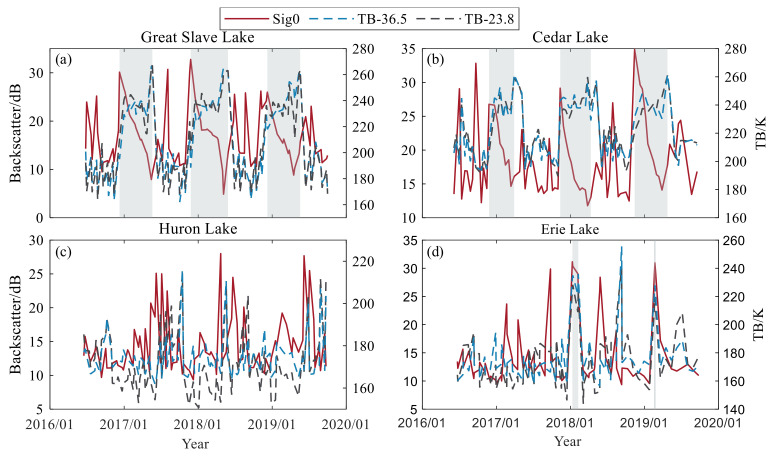
Time series variation of backscatter coefficient and brightness temperature during 2016–2019. (**a**) Great Slave Lake, (**b**) Cedar Lake, (**c**) Lake Huron, (**d**) Lake Erie. The gray background shading represents the presence of lake ice.

Figure 8 shows the time series of backscatter coefficients and brightness temperatures for the four lakes from June 2016 to October 2019. Our algorithm determined the freeze and melt dates of Great Slave Lake from 2016 to 2019 as 9 December, 25 November, 3 December, 15 May, 28 May, and 14 May, respectively. The lake surface is lake ice during each freeze date and melt date cycle. According to the Canadian Cryosphere Observation Network (https://ccin.ca/ccw/lakeice/current/monitoring, accessed on 25 June 2022), the ground-measured freezing days are 02 December, 17 November, and 29 November, and the melting days are 19 May, 1 June, and 24 May. The current state of the lake surface estimated from the backscatter coefficient and brightness temperature is consistent with actual observations from ground stations. In addition, several small peaks in the backscatter coefficient time series may occur during the ice growth period, such as Great Slave Lake during the 2018 ice season and Cedar Lake during the 2016 ice season. The peaks are caused by temporary changes in lake surface characteristics, such as warmer temperatures in early spring that cause some lake ice to melt during the day and refreeze later at night when the temperature drops.

**Figure 9:**
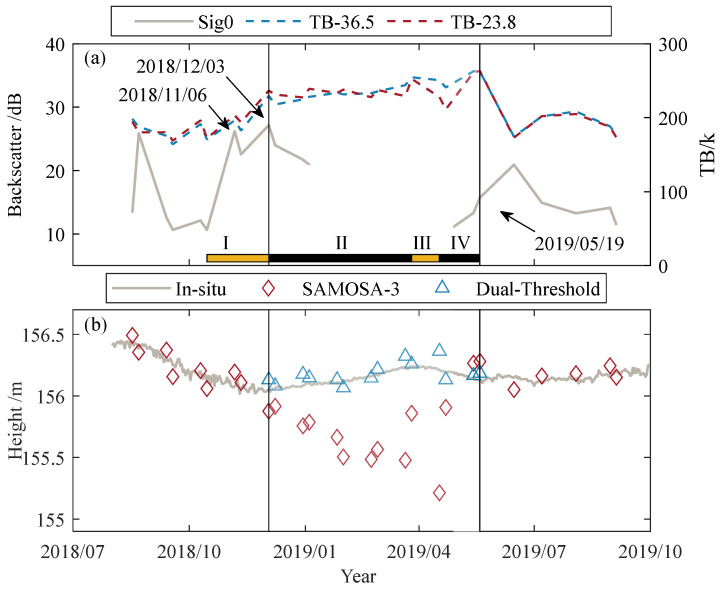
Consistency of backscattering coefficient and changes in brightness temperature and water level deviation in Great Slave Lake from 2016 to 2017. (**a**) Time series of brightness temperature and backscatter coefficient, The four line segments (I, II, III and IV) in the picture correspond to the four stages of icing. (**b**) water level estimated using the corresponding time retrospective performance analysis of the dual-threshold algorithm.

Figure 9 shows that during the ice period on Great Slave Lake in 2018, the time series of the backscatter coefficient exhibits two peaks within a short period in winter. The moving average brightness temperature difference for the first and second peaks is −18.60 K and −20.94 K, respectively. The difference of the second peak was smaller, and 3 December 2018 was identified as the freezing date of the lake ice. As lake ice grows progressively, volume scattering increases within the lake ice, and the influence of liquid water under the ice is reduced, resulting in a decrease in the backward scattering coefficient. The brightness temperature increases steadily at this time because the thicker ice reduces the influence of the low-emissivity (radiatively cold) liquid water beneath the ice and emits its microwave energy [74]. The backscatter coefficient gradually increases as the temperature increases and the lake ice melts. The peak of the backscatter coefficient in spring is sought and used as the lake ice melt point. If no peak is observed in the spring, the day the lake ice melts is determined based on the moving average brightness temperature difference. For example, there was no peak in spring 2019; therefore, the moving average temperature difference on 19 May 2019, which was calculated to be 37.53 K, the maximum over some time, was used to determine the end of the lake ice period. As shown in Figure 9b, there is a significant deviation between the water level estimated using the SAMOSA-3 algorithm and the measured water level in winter. In contrast, the dual-threshold algorithm estimates that the water level is consistent with the measured water level. The timing of the deviation is consistent with the dates of lake ice freezing and melting determined above. The Canadian Cryosphere Information Network observed the freezing and melting dates to be 7 December and 24 May, respectively. The freezing and melting dates judged based on the backscatter coefficient and brightness temperature changes are within the actual observation results of the ground station, and the lake surface type can be effectively determined.

### 4.3. Performance of the Dual-Threshold Algorithm

**Figure 10:**
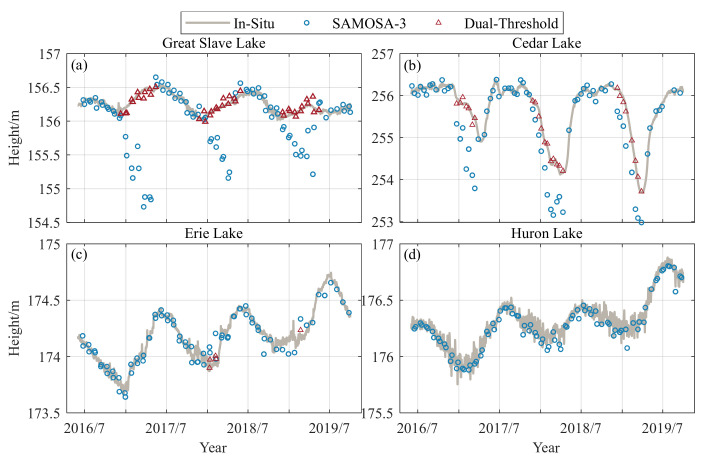
Comparing the water level obtained using the dual-threshold algorithm and the measured water level obtained using the SAMOSA-3 retracker. (**a**) Great Slave Lak, (**b**) Cedar Lake, (**c**) Lake Erie, (**d**) Lake Huron.

**Figure A1:**
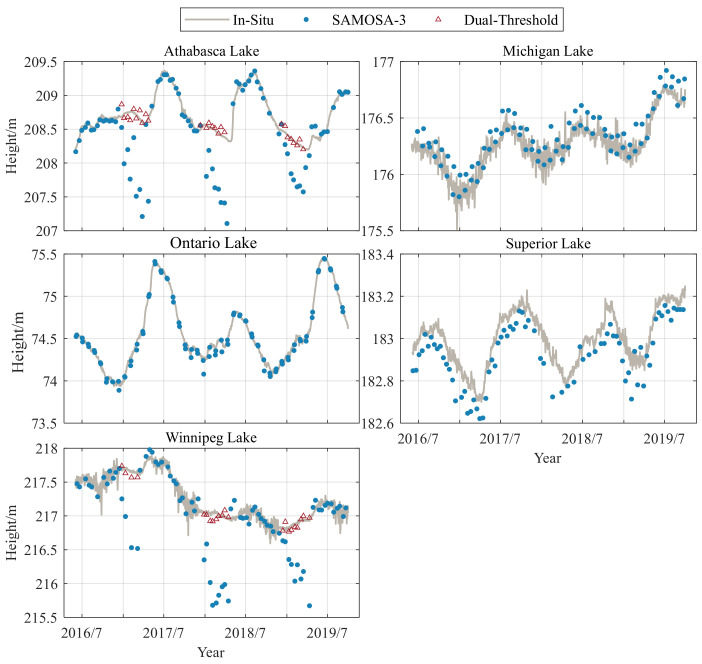
Comparison of water level time series obtained using dual-threshold algorithm and SAMOSA-3 retracker with in situ water level measurements.

Figure 10 shows the time series of water levels obtained for Great Slave Lake, Cedar Lake, Lake Erie, and Lake Huron from June 2016 to October 2018 using the dual-threshold retracking algorithm and the SAMOSA-3 (see Appendix A Figure A1 for the remaining lakes). Compared to measured water levels, the maximum deviations of SAOMOSA-3 for Great Slave Lake, Cedar Lake, and Lake Erie are 1.72 m, 1.66 m, and 0.25 m, respectively. The maximum deviations of dual-threshold for the three lakes are 0.12 m, 0.02 m, and 0.12 m, respectively. The SAMOSA-3 can obtain water level estimation results regardless of lake conditions. However, when the lake is covered by ice, the water level estimated using SAMOSA-3 deviates significantly from the measured water level. The accuracy of the lake water level estimated using the dual-threshold retracking algorithm in this paper during freezing is comparable to the accuracy of the open lake water level estimated using the SAMOSA-3 tracking algorithm, demonstrating the reliability of the algorithm. During winter, the Great Lakes may only have a thin layer of lake ice, which does not produce a double-peaked waveform. The returned waveform is still a single narrow sharp peak that still meets the standard retracking algorithm developed for SAR waveforms.

To better demonstrate the performance of the dual-threshold, the complete water level series and the measured values for three ice seasons from June 2016 to October 2019 are compared separately. Table 5 shows the comparative analysis of water levels obtained using the dual-threshold, the SAMOSA3, and the measured water levels for the nine lakes studied. When the data field in the dual-threshold column is “/”, it indicates no ice cover during the three years. When the lakes in Canada were covered with ice, the accuracy of dual-threshold was significantly higher than that of the SAMOSA-3, especially for Great Slave Lake, where R improved from 0.08 to 0.92, the bias decreased from −25.58 cm to 0.37 cm, and the RMSE decreased from 45.75 cm to 5.90 cm. For Lake Erie, R remains unchanged, the bias decreases from 8.58 cm to 7.40 cm, and the RMSE decreases from 6.89 cm to 5.01 cm. The slight improvement is because only two small ice epochs were observed near the selected hydrological station, and the ice epoch had little influence. These results demonstrate that the dual-threshold algorithm can provide continuous and stable water levels with sufficient accuracy for lakes.

## 5. Conclusions and Outlook

For lakes covered by ice in winter, there is a significant deviation between the water level inverted by the standard retracking algorithm and the measured data at the hydrological station. The thickness and stability of lake ice are critical to safe ice activities. Incorrect water level estimates can lead to inaccurate estimates of lake ice melt and freeze timing, increasing the risk of on-ice activities. Water level is essential in meteorological and climate studies, especially concerning lake effect and ice formation. More than accurate water level estimates can lead to meteorological and climate change misunderstandings. To improve the accuracy of water level inversion, it is necessary to determine the current lake surface type. This paper combines the changes in the backscattering coefficient and brightness temperature to make the judgment. The judgment results are consistent with the ground measurement results. Additionally, this article proposes a double-threshold algorithm based on the bimodal correction algorithm. The algorithm overcomes the shortcomings of the double-peak correction algorithm, cannot judge multi-peak waveforms, and requires water level deviation correction. As a result, more accurate retracking points and high-precision water level estimation are provided. In the presence of lake ice on Lake Athabasca, Cedar Lake, Great Slave Lake, Lake Winnipeg, and Lake Erie, the RMSE of water levels estimated using the SAMOSA-3 is 39.58 cm, 46.18 cm, 45.75 cm, 42.64 cm, and 6.89 cm. The RMSE of the Dual-threshold algorithm is 6.75 cm, 9.47 cm, 5.90 cm, 7.67 cm, and 5.01 cm. The results show that the dual-threshold algorithm has significantly higher accuracy than the SAMOSA-3 when the lake is ice-covered. Therefore, the dual-threshold algorithm can provide a highly accurate water level time series for ice-covered lakes at high latitudes. It is of positive significance for long-term water level monitoring and management of lakes lacking hydrological stations in mid- and high-latitude areas.

To further improve the accuracy of the dual-threshold algorithm, we can remove the interference waves caused by complex terrain near the shore and explore better and more automatic methods for distinguishing lake ice. This approach is expected to allow for the application of the algorithm to different satellites, enabling long-term, continuous, and high-accuracy water level monitoring with an improved temporal and spatial resolution of middle and high-latitude ice-covered lakes.

## Figures and Tables

**Table 1:** Hydrological station, winter ice cover, and Sentinel-3 ground track numbers of the case study lakes.

Lake Name	Country	Lon (°)	Lat (°)	Area (km^2^)	Winter Ice Cover	Sentinel-3 Tracks	Distance (km)
1. Great Slave	Canada	−114.37	62.09	27,816	Fully	37/681	21.2
2. Athabasca	Canada	−100.96	59.10	7782	Fully	54/493	8.8
3. Cedar	Canada	−100.14	53.33	2817	Fully	109/681	21.0
4. Winnipeg	Canada	−97.25	52.12	24,514	Fully	224/521	16.6
5. Superior	Canada/USA	−88.23	47.72	81,936	Partly	166/549	16.6
6. Huron	Canada/USA	−82.21	44.78	59,757	Partly	422/577	16.7
7. Ontario	Canada/USA	−77.77	43.85	19,329	Partly	107/148	11.7
8. Michigan	USA	−87.09	43.84	57,399	Partly	662/735	12.1
9. Erie	Canada/USA	−81.16	42.25	25,691	Partly	576/649	9.1

**Table 2:** Basic parameters for hydrological stations located on lake shores for accuracy verification.

Station Name	Station Code	Lat (°)	Lon (°)	Lake Name	Vertical Datum
Yellowknife Bay	07SB001	62.45	−114.35	Great Slave	CGVD28
Cracking Stone	07MC003	59.38	−108.89	Athabasca	CGVD28
Oleson Point	05KL005	53.32	−100.28	Cedar	CGVD28
Mission Point	05SG001	53.19	−99.21	Winnipeg	CGVD28
Grand Marais	9099090	47.75	−90.34	Superior	IGLD85
Harbor Beach	9075014	43.85	−82.64	Huron	IGLD85
Olcott	9052076	43.34	−78.73	Ontario	IGLD85
Calumet Haber	9087044	41.73	−87.54	Michigan	IGLD85
Fairport	9063053	41.76	−81.26	Erie	IGLD85

**Table 3:** Geophysical corrections from the Sentinel-3 Level 2 products.

Correction	Level 2 Products	Model	Range of Correction
Ionosphere	iono_cor_gim_01_ku	GIM	0–50 cm
Dry troposphere	mod_dry_tropo_cor_meas_altitude_01	ECMWF	1.7–2.5 m
Wet troposphere	mod_wet_tropo_cor_meas_altitude_01	ECMWF	0–50 cm
Polar tide	pole_tide_01	Historical pole location	±10 cm
Solid earth tide	solid_earth_tide_01	Cartwright model	±50 cm

**Table 4:** The R, bias, and the RMSE between the SRAL SAR lake level estimates and the in situ gauge water level. (If there is | after the lake name, there is a glacial period for the surface lake, the second line shows the result after excluding the glacial period data).

Lake Name	R			Bias (cm)			RMSE (cm)		
	OCOG	Ice Sheet	SAMOSA-3	OCOG	Ice Sheet	SAMOSA-3	OCOG	Ice Sheet	SAMOSA-3
Great Slave|	0.10	0.03	0.08	11.96	−16.55	−25.58	36.71	51.77	45.75
	0.93	0.96	0.95	27.26	9.23	0.49	6.30	4.85	4.84
Athabasca|	0.83	0.74	0.77	15.78	−13.72	−23.12	25.12	40.20	39.58
	0.98	0.98	0.98	26.50	7.96	0.12	8.18	5.23	6.56
Cedar|	0.92	0.91	0.92	−1.97	−24.39	−30.15	36.06	47.14	46.18
	0.97	0.97	0.97	17.58	5.84	0.2	9.64	9.63	9.25
Winnipeg|	0.73	0.68	0.71	9.60	−17.30	−23.41	34.92	46.26	42.64
	0.96	0.92	0.92	26.98	7.78	0.18	8.8	7.78	8.02
Superior	0.95	0.96	0.96	28.10	5.52	−3.28	4.5	3.77	4.46
Huron	0.97	0.99	0.99	30.07	6.64	−2.23	5.16	3.51	3.78
Ontario	0.99	0.99	0.99	31.90	7.27	−1.64	2.93	3.08	3.45
Michigan	0.96	0.97	0.97	37.55	13.65	4.20	6.38	4.65	5.34
Erie|	0.98	0.96	0.95	39.82	17.47	8.58	5.84	5.94	6.89
	0.98	0.98	0.98	40.88	16.61	7.40	5.26	4.89	5.01
Mean *				33.70	9.94	2.61	4.85	3.98	4.28

* The mean of the bias and RMSE are calculated only over 5 lakes, including the Great Lakes (Superior, Michigan, Huron, Erie, and Ontario).

**Table 5:** Comparative analysis of water levels obtained using Dual-Threshold and SAMOSA-3 retracker in 9 lakes and the in situ gauge water level.

Re-Tracker	SAMOSA-3			Dual-Threshold		
Lake Name	R	Bias (cm)	RMSE (cm)	R	Bias (cm)	RMSE (cm)
Great Slave	0.08	−25.58	45.75	0.92	0.37	5.90
Athabasca	0.77	−23.12	39.58	0.98	0.27	6.75
Cedar	0.92	−30.15	46.18	0.99	0.46	9.47
Winnipeg	0.71	−23.41	42.64	0.97	0.37	7.67
Superior	0.96	−3.28	4.46	/	/	/
Huron	0.99	−2.23	3.78	/	/	/
Ontario	0.99	−1.64	3.45	/	/	/
Michigan	0.97	4.20	5.34	/	/	/
Erie	0.98	8.58	6.89	0.98	7.40	5.01

## Data Availability

Data are contained within the article.
